# Wood–Plastic Composites: Manufacturing, Rheology and Processing and Process Modeling

**DOI:** 10.3390/ma18174042

**Published:** 2025-08-28

**Authors:** Krzysztof Wilczyński, Kamila Buziak, Adam Wilczyński

**Affiliations:** 1 The Faculty of Mechanical and Industrial Engineeering, Warsaw University of Technology, 02-524 Warsaw, Poland; kamila.buziak@pw.edu.pl; 2 PolymerSoft, 85-063 Bydgoszcz, Poland

**Keywords:** wood–plastic composites, rheology, viscosity, yield stress, slip effects, process modeling, extrusion, injection molding

## Abstract

Wood–plastic composites (WPCs) are polymeric materials, usually thermoplastic, filled with wood flour or fibers. They are relatively durable and stiff and resistant to water. They are also, importantly, relatively cheap compared to materials with similar properties. The WPCs market has grown significantly in recent years, mainly thanks to the increasing construction and automotive markets. Currently, the global WPCs market is forecasted to reach about USD 15 billion by 2030, increasing at an impressive compound annual increase rate of about 12% until 2030. There are some review articles on WPCs written from many different points of view, e.g., the type of materials used (polymers, fillers, auxiliaries), the method of manufacturing and processing, processing properties (thermal and rheological) and functional properties, methods of designing composite products and designing (modeling) forming processes. In this article, we will summarize these different points of view and will present a thorough literature review of rheology and material processing, and more specifically, the modeling of WPCs processing. This work will be presented in relation to state-of-the-art research in the field of modeling the processing of other polymeric materials, i.e., standard (neat) polymers and polymer blends. The WPCs’ processing is significantly different from that of standard plastics due to the differences in thermo-rheological properties, diverse structures, etc. So far, the global WPCs processing models have only been developed for both gravity-fed and starve-fed single-screw extrusion. The models for twin-screw extrusion, both co-rotating and counter-rotating, as well as for injection molding, have still not been developed. WPCs show a yield stress and wall slip when extruding, which must be considered when modeling the process. As the slippage on the screw and barrel grows, the process throughput and pressure diminish, but as the slippage on the die grows, the throughput grows and the pressure diminish. As the yield stress in the screw grows, the process throughput and pressure grow, whereas as the yield stress in the die grows, the throughput diminishes and the pressure grows.

## 1. Introduction

Wood–plastic composites (WPCs) are polymeric materials, usually thermoplastic, reinforced (filled) with wood flour or wood fibers. Synthetic thermoplastics form a matrix surrounding the natural wood fillers. These matrix materials flow easily with heating up, meaning flexible processing when wood fillers are compounded with them. Thermoplastics shrink and swell, but they can be an effective barrier preventing moisture from penetrating the composite. Wood fillers are composed of natural polymers such as cellulose, etc., and have completely different properties from synthetic materials. Wood is cheaper and stronger than thermoplastics and has low density. Wood fillers do not shrink or swell significantly with temperature, but readily absorb moisture, leading to biodegradation if unprotected. WPCs are relatively durable and stiff, and resistant to water. They are also, importantly, relatively cheap compared to materials with similar properties.

Interestingly, the first wood–plastic composites were created on the basis of thermosetting polymers and appeared at the beginning of the 20th century. Bakelite, the first plastic made of synthetic components, developed by Leo Baekeland in 1907 as a combination of phenol-formaldehyde resin and wood flour, was one of the first composites of this type and was applied as a gear knob in Rolls Royce cars in 1916. At that time, natural fibers were used only as fillers for phenolic resins and unsaturated polyesters [1]. It was not until the 1990s, with the growth of environmental awareness, that wood-based composites based on thermoplastics appeared. They gradually began to attract the attention of entrepreneurs and scientists from around the world, characterized from the beginning by a rapid increase in market share. The 21st century brought exceptional development of this industry. This evolution was possible thanks to the combination of knowledge and experience of the plastics industry with the perspective and resources of the forestry industry. The development of such natural fiber-filled composites is of high importance for the conservation of forests and the use of natural fiber waste, and is also compatible with the circular economy.

The WPCs market has grown significantly in recent years, mainly due to the growing construction and automotive markets [2,3]. For example, road transport is responsible for greenhouse-gas emissions and contributes to global warming. Manufacturers are striving to reduce vehicle weight to lower CO_2_ emissions, which can be achieved by using composites with natural reinforcements (WPCs-based). Increasing environmental pressures and new opportunities for using natural composite materials are pushing manufacturers to use these composites, aiming to reduce the use of costly and non-recyclable reinforcements, such as glass fiber.

Currently, more than 350 million tons of plastic waste is generated worldwide each year. Estimates suggest that this amount will triple by 2060. Therefore, the global economy is focusing on sustainable development and saving limited resources such as oil and forest products. This trend has contributed to the development of composite materials made of plastics with a significant share of forest waste [4,5,6], which are becoming competitive with regard to synthetic or inorganic fillers, mainly due to their lower dependence on fossil sources and better environmental impact [7,8,9,10].

Currently, the global WPC market is forecasted to reach USD 15.41 billion by 2030, increasing at an impressive compound annual increase rate (CAGR) of 11.6% from 2023 to 2030 [11]. IMARC Group forecasts the WPC market will achieve USD 9.3 billion by 2028 with a compound annual increase rate of 11.2% from 2023 to 2028 [12]. According to Research Nester, this value could increase to USD 20 billion by 2035 [13].

The largest market share in 2019 was held by North America. The main application of WPCs in this region is construction. The Asian market, especially China and India, is also a significant player, generating a significant part of the global production and innovation in the field of WPCs. The main shareholders of the European WPCs market are Germany and the United Kingdom.

The manufacturing process of wood–plastic composites embraces two stages, i.e., compounding and forming. The process of compounding of wood and other additives is carried out to incorporate them into a molten thermoplastic matrix to produce a homogeneous composite material. And, this composite is then formed into a product mainly by extrusion and injection molding.

The polypropylene (PP), high density polyethylene (HDPE) and poly(vinyl chloride) (PVC) are the basic thermoplastics for WPCs. Various forms of polyethylene (HDPE, LDPE, LLDPE etc.) are represented in WPCs at 65%, poly(vinyl chloride) (PVC) at 15% and polypropylene (PP) at 10%. The production of eco-composites using biopolymers as a base is also interesting [14,15].

The issues related to wood–plastic composites have been discussed in several fundamental books, e.g., by Mohanty et al. [16], Klyosov [17] and Oksman Niska and Sain [18] or review articles, e.g., of Bledzki et al. [19,20], Faruk et al. [21], Zajchowski and Ryszkowska [22] or Stewart [23]. Klyosov’s book [17] was the first to systematize the principles underlying the composition of wood–plastic composites and their properties under real conditions. The important book of Oksman Niska and Sain [8] reviewed the production of wood–plastic composites, how to evaluate and improve their properties and the range of their applications. The book covers key aspects of production, including raw materials, production technologies and interactions between wood and synthetic polymers. Building on this foundation, the book discusses mechanical and other properties such as durability, creep behavior and processing efficiency.

There are some review articles on wood–plastic composites written from many different points of view, e.g., the type of materials used (polymers, fillers and auxiliaries), the method of manufacturing and processing, processing properties (e.g., thermal and rheological) and functional properties, methods of designing composite products and designing (e.g., modeling) forming processes. In this article, we will summarize these different points of view and will present a thorough literature review of rheology and material processing, and more specifically, the modeling of wood composites processing. This work will be presented in relation to state-of-the-art research in the field of modeling the processing of other polymeric materials, i.e., standard (neat, pure) polymers and polymer blends.

## 2. Materials, Manufacturing, Processing

Bledzki et al. [20] were the first to present the issues of WPCs processing. They pointed out that the surfaces of polymer matrix and wood fillers should be modified to build composites of high strength and long service life. However, this is complex and dependent on each material composition and process conditions.

Chan et al. [24] presented a review on composites made of biodegradable thermoplastics. They concluded that the properties of composites are dependent on the polymer matrix and wood-filler type and content, compatibilization technique used and processing parameters. The extent of interfacial adhesion and filler morphology were identified as the primary factors controlling the composite properties. Consistency was achieved throughout the literature on the reinforcing effects of wood fillers. A general trend of improving composite stiffness with an increase of wood content was observed. The strength of the composite is dependent on the adhesion between a matrix and fillers which is dependent on the polymer nature. The strength of hydrophobic polymer-based WPCs diminishes with the growth of wood content, but the strength of hydrophilic polymer-based WPCs grows. The paper indicates the need for further research, including establishing basic relations between quantitative interfacial adhesive forces and final material characteristics of composites.

Khan et al. [25] presented a review on the material characterization, processing and applications of wood–plastic composites. They concluded that wood–plastic composites with a content of 20–30% of smaller wood particles showed the best mechanical properties. They added that several techniques may be used to improve the mechanical properties of wood–plastic composites, e.g., hybridization of different types of wood fillers and chemical pretreatment as well as addition of compatibilizers. They also remarked that the mechanical properties of 3D printed wood–plastic composites were inferior to those of virgin polymers due to the low pressure in production.

Yadav et al. [26] reviewed the recent advances in wood–plastic composites. They discussed various methods of processing the polymer composites and pointed out the advantages of extrusion and injection molding and the disadvantages of other techniques. They drew attention to the issue of using nanofillers in polymer composites, e.g., nanoclays, which are eco-friendly, easily available in large amounts and cheap fillers.

A very extensive review was given by Ramesh et al. [27] who discussed the material aspects of wood–plastic composites and the additives used. Among others, they indicated the use of bio-based adhesives such as starch or lignin, which may be considered as an environmentally friendly option to conventional synthetic adhesives.

Elsheikh et al. [28] presented a comprehensive review of WPCs manufacturing techniques, among them, pre- and post- processing ones. Chemical, mechanical and thermal techniques were discussed. Extrusion and injection molding were considered as the basic manufacturing techniques.

Sun et al. [29] reviewed the general trend and development history of the composites reinforced with natural fiber and analyzed their role in the global polymer market. They also pointed out the most important research results in this field, and the main areas of their industrial applications were also summarized. The development prospects of natural fiber composites were also indicated.

A very recent review was presented by Mital’ová et al. [30]. The authors, among others, concluded that the use of natural reinforcements can reduce the carbon footprint, and biodegradable materials can also be used as WPCs matrices, e.g., based on polylactic acid (PLA), polyhydroxyalkanoates (PHA, PHB and PHBV), starch derivatives and cellulose, etc.

Olakanmi and Strydom [31] explained some of the material and processing issues (poor bonding and degradation at the wood fiber and polymer interface, fiber damage during processing, etc.) that influence WPCs’ interface and functional performances and proposed appropriate remedies.

The different sources of materials used for the manufacturing of wood–plastic composites have been discussed by Yama et al. [32], Clemons [33], Deka and Maji [34], Martins et al. [35], Krause et al. [36], Asim et al. [37], Bollakayal et al. [38] and Leao et al. [39]. For example, Leao et al. [39] presented the present scenario and future scope of the use of wood waste in WPCs. Han et al. presented advanced studies on cellulose nanofibers [40,41].

Wood–plastic composites are mainly manufactured and processed by extrusion and injection molding. Various processing issues were discussed by Gonzalez and Myers [42], Clemons and Ibach [43], Błędzki and Faruk [44,45], Stark [46], Burgstaller [47], Nourbakhsh and Ashori [48], Migneault et al. [49,50], Gacitua et al. [51], Peltola et al. [52], Shahi et al. [53], Gardner et al. [54], Teuber et al. [55], Wiedel et al. [56] and Raj [57]. For example, Raj [57] provided a literature review of WPCs that have been developed by various authors who have worked with different polymers reinforced with versatile wood flours using various processing methods.

Specific issues related to WPCs’ extrusion were discussed by Nygård et al. [58], Guo et al. [59], Crookston et al. [60], Vandi et al. [61] and Wang [62]. For example, Nygård et al. [58] performed a comparative study of extrusion-based composites reinforced with wood powder and pelletized wood fibers. A significant improvement in mechanical properties was observed for the wood-fiber-reinforced composites compared to the wood-powder-filled composites.

Specific issues related to WPCs injection molding have been discussed by Moriwaki [63], Bledzki and Faruk [64,65], Yoon et al. [66], Behravesh et al. [67], Azaman et al. [68], Gong et al. [69], Ansari et al. [70], Azad and Shahrajabian [71], Guo et al. [72,73,74,75], Moritzer and Richters [76] and Rabbi et al. [77]. For example, Rabbi et al. [77] reviewed bio-composites fabricated by the injection-molding process. Technical characteristics of injection-molded natural fiber reinforcement-based composites, their type and compounding process prior to molding were discussed.

## 3. Rheology

**Figure 1:**
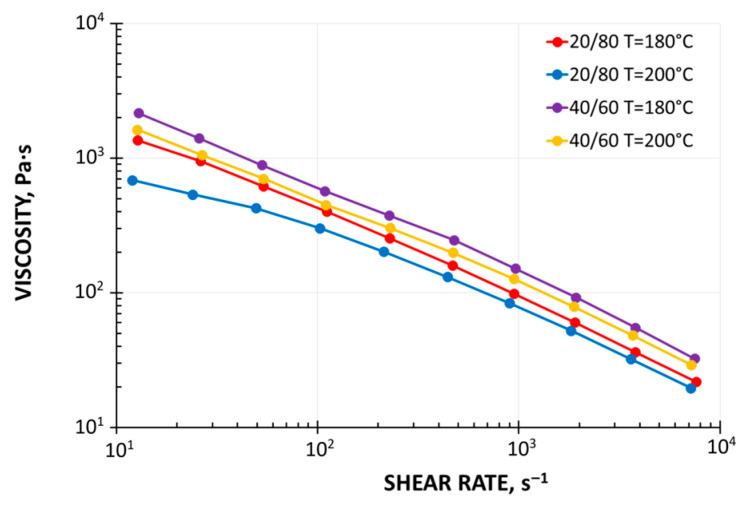
Viscosity curves of wood–plastic composites: WPC 20/80 ForMi GP20 and WPC 40/60 ForMi EXP40 (manufactured by The Biofore Company UPM, Helsinki, Finland) [88].

It is now known that wood–plastic composites have non-Newtonian, pseudoplastic and viscoelastic properties. Their viscosity diminishes with a growth of shear rate and temperature and grows with the growth of filler amount (Figure 1). It is also known that their elasticity diminishes with the growth of filler amount [16,17,18,78,79,80].

Wood–plastic composites show a yield stress and wall slip when extruding [78,81,82,83]. The slip velocity depends on the filler amount and shear rate. With the growth of shear rate, the slip velocity grows, leading to the plug flow (Vlachopoulos and Hristov [83]). Moreover, increasing the filler amount promotes the plug flow (Zolfaghari et al. [84]). Recently, Lewandowski and Wilczyński [85,86] conducted extensive FEM studies on polymer extrusion with yield stress and slip effects.

Laufer et al. [87] carried out an interesting study for WPCs and found that the shear thinning of WPCs can be described by a power law model and the consistency index correlates well with the filler volume fraction. The authors introduced the concept of an interaction exponent (defined as the ratio of flow exponent of WPC and flow exponent of polymer matrix) for interparticular interaction effects of wood particles, which shows a good correlation with the consistency index. The flow behavior of the material was described using the concept of shear-stress-equivalent inner shear rate, and the shear thinning behavior of WPCs were estimated with good accuracy, taking into account the volume fraction of wood.

A valuable review article on the rheology of wood–plastic composites and its implications for processing was recently presented by Mazzanti and Mollica [89,90]. All the articles cited by the authors agreed on the non-Newtonian behavior of these fluids, e.g., [82,87,91,92,93,94,95,96], with the viscosity being shear-rate dependent and decreasing with shear rate. This has been explained by the fact that the secondary bonds between macromolecules are constantly being broken and reformed during the flow. At higher shear rates, these do not have enough time to reform, leading to a reduction in viscosity. The fiber orientation phenomena also play a role in this phenomena. The authors concluded that the wood content mostly influences the rheological properties of WPCs. Both the shear viscosity and complex viscosity are relatively high and decrease with the shear rate and frequency. However, the important conclusion is that more valuable information about processing can be obtained from the measurement of slip velocity, which may well be interpreted as being more important than viscosity since it is the single factor that mostly influences the possible defects of the final product.

Several papers have studied the effect of wood-filler content on viscosity [78,82,87,91,93,95,97,98,99,100,101,102,103,104,105]. All agreed that viscosity grows with filler content, independently of other factors. Wood content also has a major effect on the slip behavior at the wall, with the slip velocity growing with the content of wood filler [79,82,91,93,98,100,103,106,107]. The slip velocity is of the order of 1–10 mm/s at a shear stress of the order of 200 kPa. Many studies have investigated the effect of particle size [78,92,94,95,100], showing that as the wood particle size decreases, the apparent viscosity increases. The authors summarized the influence of wood filler (amount, type and morphology of fillers and their pre-treatment), the influence of polymer matrix and temperature and the influence of additives, as well as the issues of rheological modeling. They also concluded that capillary and parallel plate rheometry are useful for studying the rheological behavior of wood–plastic composites. Parallel plate rheometry is particularly useful for studying the composite phase and obtaining structural material information, while capillary viscometry is convenient for understanding the final manufacturing. An important conclusion is that when using rotational rheometers at lower frequencies, the flow properties are dependent on the filler behavior, whereas at high frequencies they are dominated by the matrix. An important conclusion is that wall slip can actually be understood as being more important than viscosity, since it affects the quality of the final product.

Other contributions in the field of WPCs rheology have been presented by, among others, Ares [108] who concluded that recycled polypropylene is suitable for the production of wood composites, Ewurum [109], who evaluated the rheological properties of mixed plastic waste (MPW), Talcott [110], who evaluated the use of waste natural fibers without chemical treatment and compared them to a commercial wood fiber for use in WPCs, as well as Feng [111] who investigated the extrusion processibility of high density polyethylene (HDPE) composite by analyzing the torque rheological behavior of the material.

## 4. Process Modeling

### 4.1. Polymer Extrusion

It is well known that the basic technology for processing of wood–plastic composites is extrusion. The latest state of knowledge in polymer-processing modeling, including extrusion, has been recently presented by Wilczyński [112]. However, the fundamental monograph on polymer extrusion, which can be called the “Bible of extrusion”, was written by Tadmor and Klein [113]. Ariffin and Ahmad [114], as well as Wilczyński et al. [115], reviewed some important aspects of polymer extrusion modeling.

Knowledge of the material flow mechanism in the extruder is the fundamental for process modeling. The flow of molten materials is fairly well described, but only for viscous materials. The flow of viscoelastic materials is much less understood. The flow of composite materials with yield stress and wall slip is also poorly recognized. Thus, it can be concluded that extrusion modeling is limited to viscous materials. When modeling the extrusion process it is important to note that the models both for a plasticating unit and for an extrusion die should be developed [103,116,117,118]. 

The transport (conveying) and melting (plasticizing, fusion) of solids are still poorly described, especially for polymer blends and composites.

**Figure 2:**
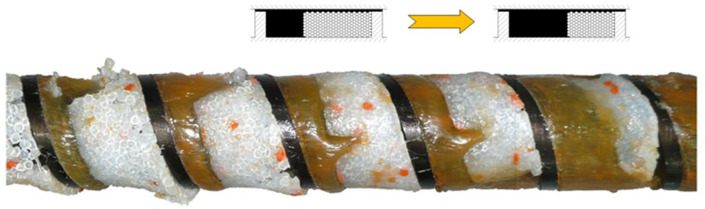
The mechanism of polymer fusion in the gravity-fed SSE-machine, (PP) extrusion [121].

Polymer melting (polymer fusion) in single-screw extruders, i.e., SSE-machines, has been widely examined. Maddock [119] and Street [120] were the first to perform fusion experiments in gravity-fed (flood-fed) extruders using the “screw pull-out technique”. They rapidly cooled the extruder, removed the screw and stripped the polymer from the screw, which was cut in a cross-section. According to these studies, a melt layer is clearly visible along the barrel and a melt pool, “a lake”, close to the active flight. Many other researchers, e.g., Wilczyński et al. [121] (Figure 2), confirmed these observations.

Tadmor et al. [113,122,123] were the first to develop a model of polymer fusion in the gravity-fed SSE-machines and, on this base, the first global model of the SSE-process. According to this model, a melt layer is formed between the barrel and the solid, and the melted polymer is scraped off by the transverse flow and accumulates during the active flight of the screw. The width of the solid bed is gradually reduced by the effect of heating from the heaters and viscous dissipation in the molten polymer. Later, several melting models and global extrusion models were developed [124,125,126,127,128].

**Figure 3:**
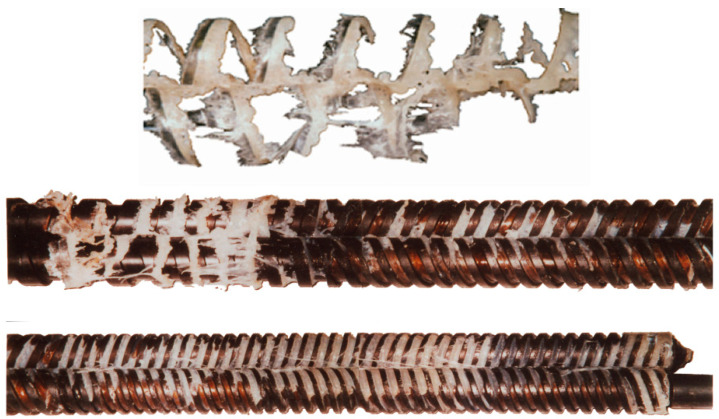
The mechanism of polymer fusion in the counter-rotating TSE-machine, (PP) extrusion [141].

In contrast to polymer fusion in SSE-machines, research on fusion in twin-screw extruders (TSE-machines), both co-rotating (CO-TSE) and counter-rotating (CR-TSE), appeared in the literature much later. Several fusion models for co-rotating TSE-machines have been developed, e.g., [129,130,131,132,133,134,135], as well as several global models, e.g., [136,137,138]. The studies on fusion in counter-rotating TSE-machines were very limited [139,140] (Figure 3). The fusion models and the global models were developed by Wilczyński et al. [141,142,143,144], Lewandowski et al. [145], Jiang et al. [146].

**Figure 4:**
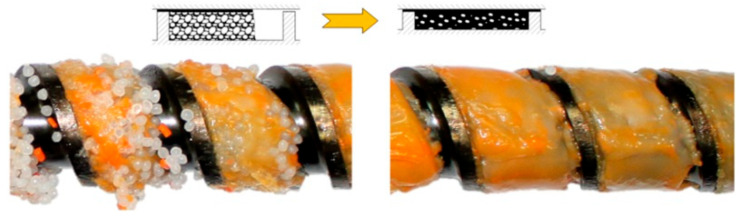
The mechanism of polymer fusion in the starve-fed SSE-machine, (PP) extrusion [121].

Studies on starve-fed (metered-fed) SSE-process are extremely limited. Recently, Wilczyński et al. [121] revealed the fusion mechanism in the starve-fed SSE-machines (Figure 4), developed a polymer fusion model [147] and finally a global process model [148,149,150].

**Figure 5:**
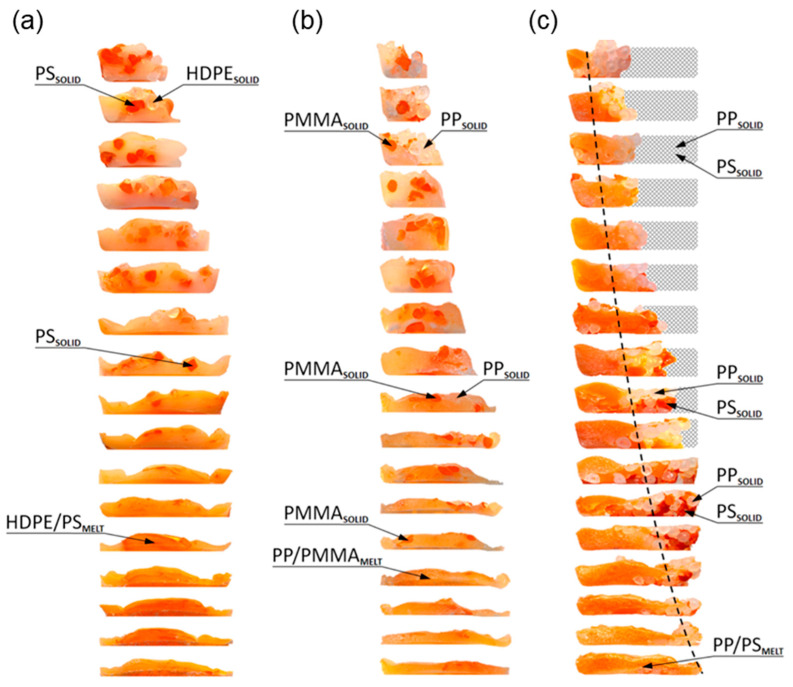
The mechanism of polymer blends fusion: (**a**) polymer blend (HDPE/PS)—starve-fed process, (**b**) polymer blend (PP/PMMA)—starve-fed process, (**c**) polymer blend (PP/PS)—gravity-fed process [140].

**Figure 6:**
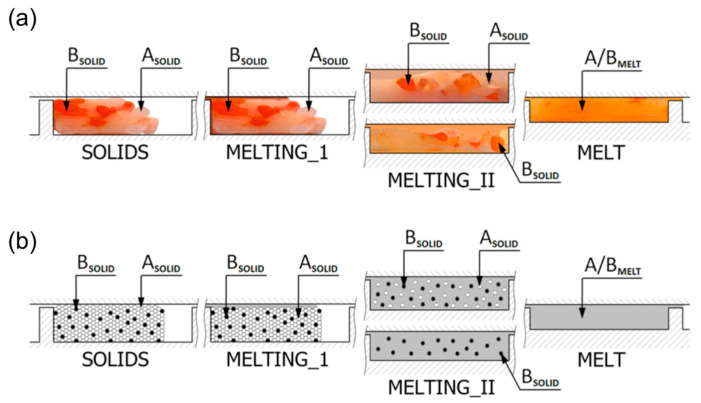
The mechanism of polymer blends fusion in the starve-fed SSE-machine: (**a**) fusion visualization, (**b**) fusion model: A—major component of polyblend (HDPE), B—minor component of polyblend (PS), A/B—polyblend (HDPE/PS), MELTING_I—fusion by heat conduction, MELTING_II—fusion by energy dissipation [140].

**Figure 7:**
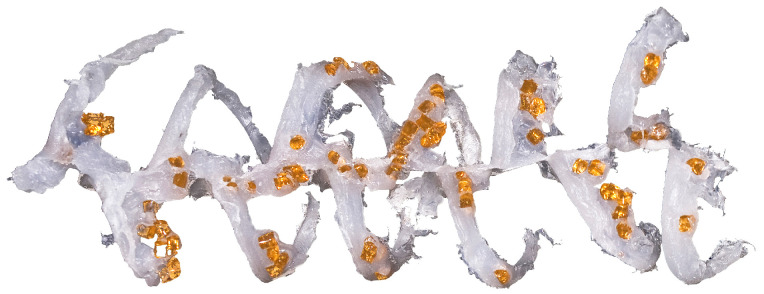
The sample of polymer blend (LDPE/PS) from counter-rotating TSE-machine: white color—major component of polyblend (LDPE), amber color—minor component of polyblend (PS) [140].

**Figure 8:**
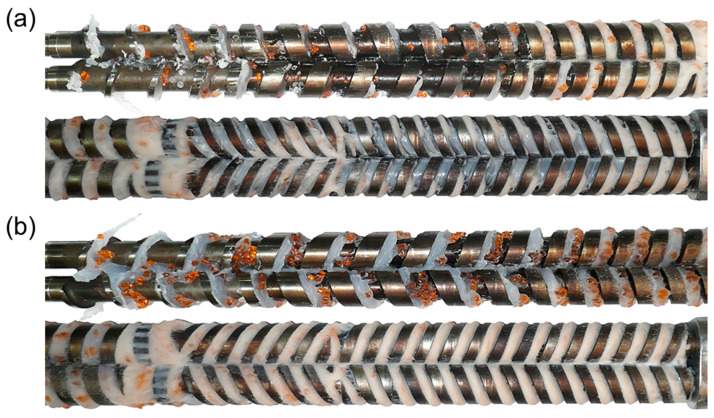
The screws from the counter-rotating TSE-machine, polymer blend (LDPE/PS) extrusion: (**a**) top view, (**b**) bottom view, white color—major component of polyblend (LDPE), amber color—minor component of polyblend (PS) [140].

Currently, polymer blends are an important group of polymeric materials. The basis for modeling the extrusion process of these complex materials is the knowledge of the material flow mechanism in the machine, i.e., solid conveying, fusion and melt flow, which is different than in the case of extrusion of neat polymers. Wilczyński et al. [151,152,153] presented different fusion mechanisms of polymer blends in the SSE-machines, both gravity-fed and starve-fed (Figure 5 and Figure 6) and developed global models of these processes using conventional and unconventional screws [152,154,155]. The authors also investigated the counter-rotating TSE-process of polyblends [140] (Figure 7 and Figure 8).

In summary, the issue of polymer extrusion modeling has been presented in review articles, e.g., [114,115,117,156,157], and fundamental monographs, e.g., [112,158,159,160].

### 4.2. WPCs Extrusion

#### 4.2.1. Gravity-Fed Extrusion

The WPCs’ extrusion is significantly different from that of neat plastics due to the differences in thermo-rheological properties of these materials and diverse structures, etc. There has been limited research on WPCs rheology and extrusion. Basic studies have been conducted by Tzoganakis and Xiao [81,161,162,163] and Walcott and Li [78,79,80], as well as by others [18,82,83,84,92].

The first models did not describe the course of WPCs’ extrusion correctly [81,161,162,163]. Tzoganakis and Xiao [81,161,162,163] studied the fusion of (HDPE)-based composites in SSE-machines. They revealed the not-fully-filled channels in the extruder. This could presumably be due to screw and die interactions and insufficient pressure generation in the machine, leading to starvation in the screw channel. The authors also observed slight segregation of the components constituting the HDPE/WPC blend [163]. Near the rear screw flight, mainly HDPE was visible, while WPC was mainly in the direction opposite the attacking flight. The authors simulated the process with a computer program (noname), but the computations of the pressure profile in the machine were not correct.

WPCs show specific surface tearing when extruding [82,83,92]. This can be eliminated, in the engineering practice by cooling the die. There have been attempts to explain the tearing phenomenon by modeling the flow in the die [92].

**Figure 9:**
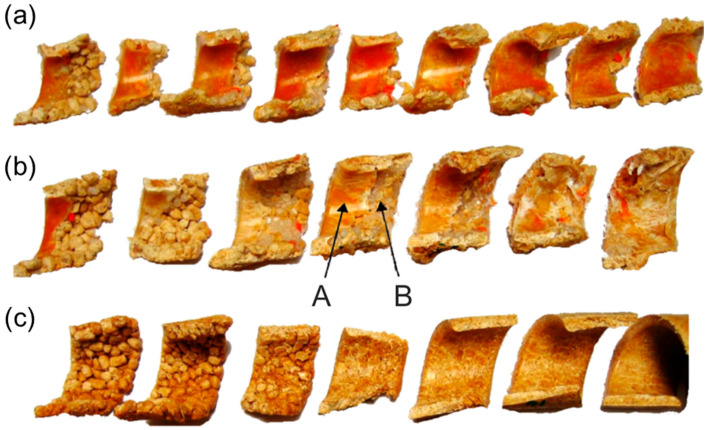
The samples of (PP)-based composites from SSE-machine: (**a**) 25% WF, (**b**) 50% WF, (**c**) 75% WF, A—molten composite, B—solid composite [164] (with permission from Int. Polym. Process. 2015, 30, 113–120, by Wilczyński, K.; Nastaj, A., Lewandowski, A., Wilczyński, K.J., Buziak, K. © Carl Hanser Verlag GmbH & Co. KG, Muenchen).

**Figure 10:**
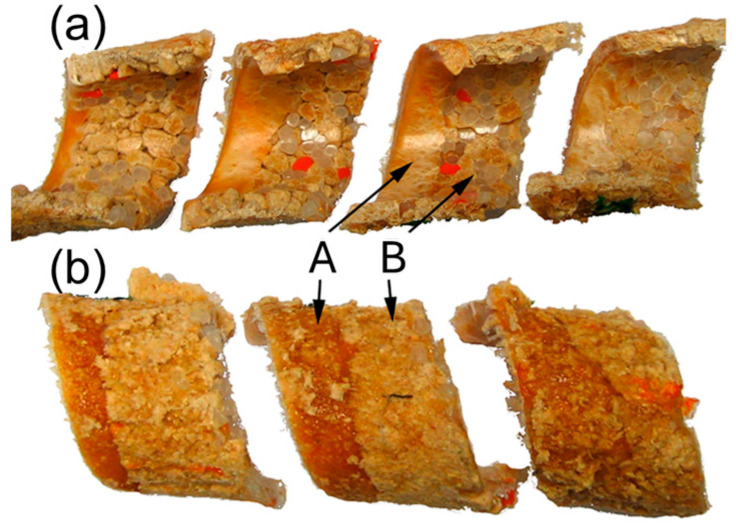
The samples of (PP)-based composites from SSE-machine (50% WF): (**a**) inner surface of the sample (screw view), (**b**) outer surface of the sample (barrel view), A—molten composite, B—solid composite [164] (with permission from Int. Polym. Process. 2015, 30, 113–120, by Wilczyński, K.; Nastaj, A., Lewandowski, A., Wilczyński, K.J., Buziak, K. © Carl Hanser Verlag GmbH & Co. KG, Muenchen).

Comprehensive studies on the extrusion of (PP)-based composites were conducted by Wilczyński et al. [164,165]. The composite conveying and fusion were strongly dependent on the WF content and extrusion conditions. The classical Tadmor fusion mechanism [113] (Figure 1) was not seen in the case of composites with more WF content. However, it was seen in the case of composites with less WF content, up to 50% (Figure 9 and Figure 10).

For a long time, there was no WPCs extrusion model describing comprehensively the solid conveying, composite fusion and molten composite flow. The state of knowledge in the field of extrusion of filled polymers was reviewed by Ariffin and Ahmad [114] and more recently, polymer extrusion modeling was reviewed by Wilczyński et al. [166].

**Figure 11:**
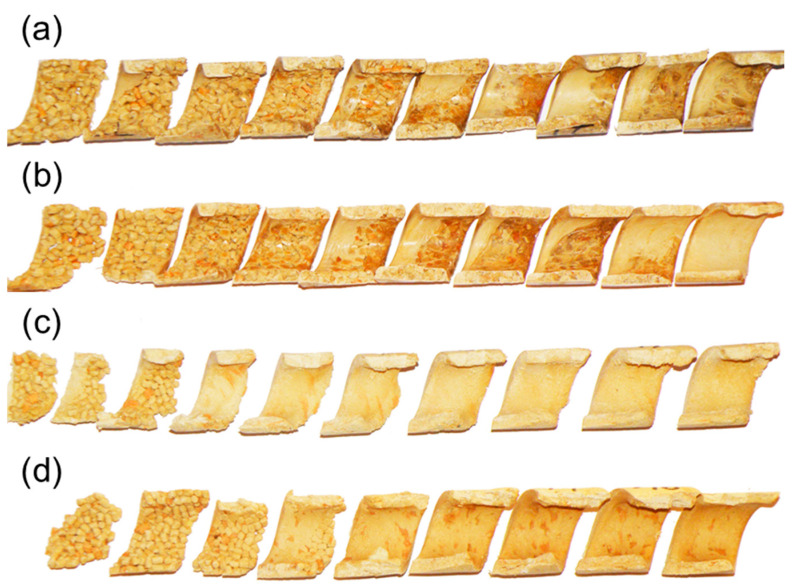
The samples of (PP)-based composites from SSE-machine: (**a**) 30% WF, (**b**) 40% WF, (**c**) 50% WF, (**d**) 60% WF [167].

**Figure 12:**
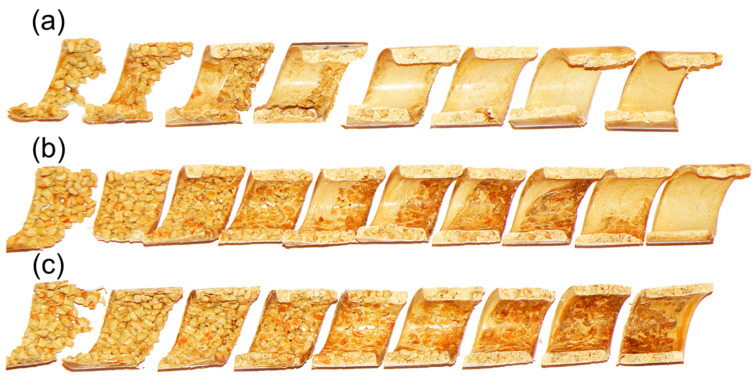
The samples of (PP)-based composites from SSE-machine (40% WF): (**a**) N_1_ = 20 min^−1^, (**b**) N_2_ = 50 min^−1^, (**c**) N_3_ = 80 min^−1^ [167].

**Figure 13:**
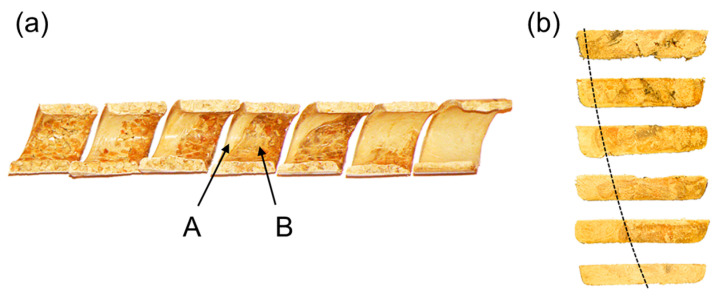
The mechanism of fusion of (PP)-based composite from SSE-machine: (**a**) inner surface of the sample (40% WF), (**b**) cross-sections of the sample (30% WF), A—molten composite, B—solid composite [167].

**Figure 14:**
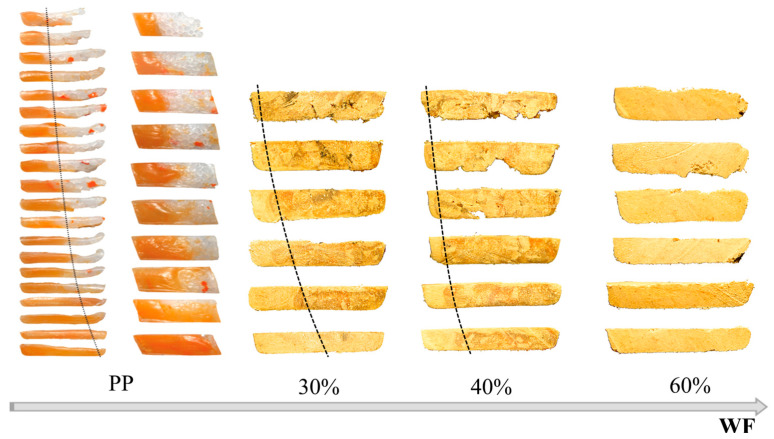
The mechanism of fusion of (PP)-based composites of various WF content, from SSE-machine, in comparison to the mechanism of fusion of neat (PP) [167].

Only recently, the authors discussed the issue of modeling the WPCs SSE-process [167]. They proposed the comprehensive model of the process and verified this model by conducting an experiment. Examples of the research results are shown in Figure 11, Figure 12, Figure 13 and Figure 14. The influence of the WF content on the composite behavior in the screw is depicted in Figure 11. It is seen that at less WF content, a Tadmor fusion mechanism occurs, but at more WF content this is not seen. This is due to the low amount of melted polymer, which is not sufficient to form a melt pool. The melted polymer penetrates the unmelted layer and the fusion is one-dimensional, from the barrel to the screw root. This is similar to the extrusion of polymers in a powder form [168].

It was observed that the screw speed strongly affects the composite behavior which is depicted in Figure 12. It can be seen here that with increasing screw speed, fusion starts later, i.e., further from the hopper and closer to the die. Fusion seems to be slower because the residence time of the material in the extruder is shorter.

The classical Tadmor fusion mechanism of WPCs in the SSE-machine is depicted in Figure 13.

Figure 14 shows the cross-sections of composites with various WF content in comparison to the cross-sections of polypropylene (PP). The similarity is clearly seen, although with increasing WF content the melt pool diminishes.

**Figure 15:**
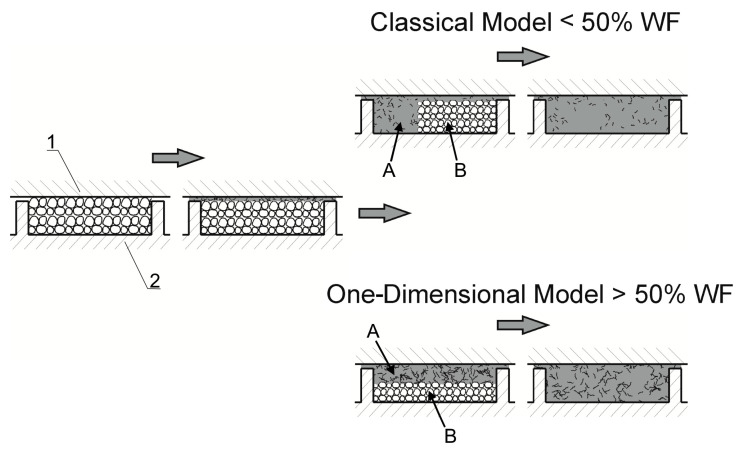
The model of WPCs fusion in the SSE-machine for various WF content: 1—barrel, 2—screw, A—molten composite, B—solid composite [167].

Based on the conducted studies, the mechanism of fusion of WPCs in the SSE-machine has been postulated, which is shown in Figure 15.

#### 4.2.2. Starve-Fed Extrusion

For a long time, there has been no research on the metered-fed WPCs extrusion (starve-fed extrusion). The only testing simulations were carried out with the use of the SSEM computer model of metered extrusion of thermoplastics [165,169].

Recently, research has been carried out on the extrusion of (PP)-based composites with a different WF amount, and a new computer model of the WPCs’ SSE-process with dosing has been built [88].

**Figure 16:**
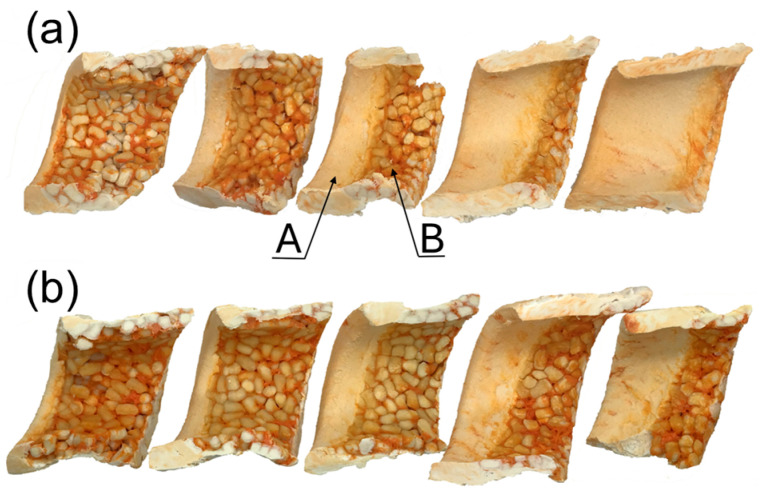
The samples of (PP)-based composites from SSE-machine (40% WF), at gravity feeding: (**a**) N_1_ = 30 min^−1^, (**b**) N_2_ = 70 min^−1^, A—molten composite, B—solid composite [88].

**Figure 17:**
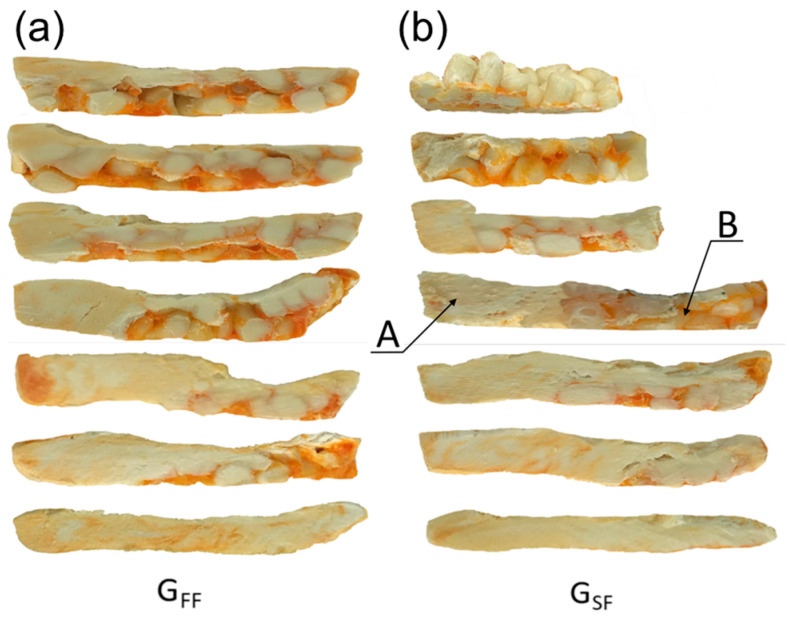
The cross-sections of (PP)-based composites from SSE-machine (40% WF), at various feeding modes: (**a**) GFF—gravity feeding, (**b**) GSF—starve feeding, A—molten composite, B—solid composite [88].

The aim of the experiment was to understand the fusion mechanism of WPCs in the SSE-machine, as well as to validate the process model. The flow mechanism at gravity feeding was compared with the flow mechanism at metered feeding. Examples of the research results are shown in Figure 16 and Figure 17. When gravity feeding (Figure 15), fusion starts later with an increase of screw speed, i.e., further from the hopper. Fusion is slower because the material residence time is shorter.

An effect of the feeding mode on the composite flow is shown in Figure 16. For the gravity feeding (GFF), the classical 2-D fusion is observed, i.e., Tadmor mechanism, whereas for the metered (starve) feeding (GSF), the conductive fusion is visible in the not-fully-filled area of the screw and classical 2-D fusion is observed in the completely filled area, which differs from the fusion of pure polymers, e.g., (LDPE), (PP), (PS), in starve-fed SSE-process where dispersed fusion is seen [121].

**Figure 18:**
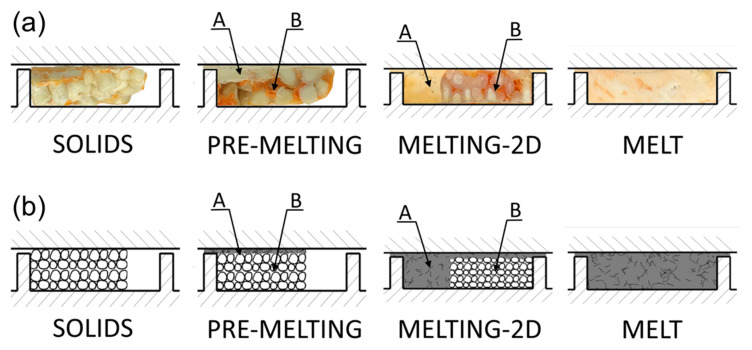
Fusion mechanism of WPCs in the starve-fed SSE-machine: (**a**) visualization of fusion, (**b**) schematic model of fusion, A—molten composite, B—solid composite [88].

Based on this experiment, fusion mechanism of WPCs in the starve-fed SSE-machine has been postulated [88], that is, fusion by conduction in the not-fully-filled area and the 2-D Tadmor mechanism in the completely filled area, which is presented in Figure 18.

#### 4.2.3. Extrusion Process Modeling

The extrusion process involves continuous co-operation of the extruder and the die, and its course depends on the mode of feeding the machine, that is, whether it is gravity (flood-fed) or metered (starve-fed) feeding, that is, with dosing. Therefore, when global process modeling, calculation schemes should be used according to the extruder feeding mode.

**Figure 19:**
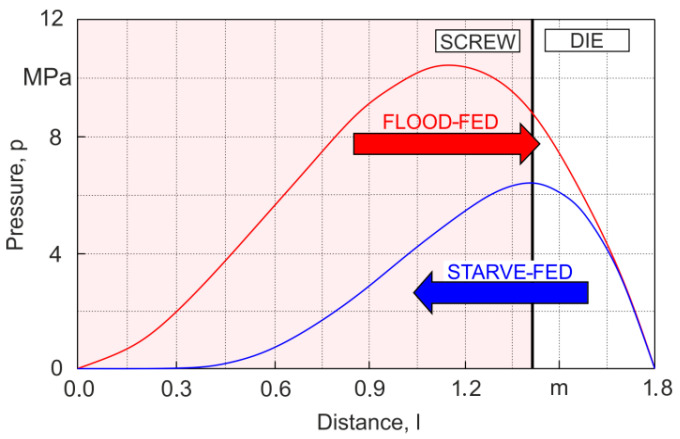
Schemes of calculations for global process modeling, a forward scheme for gravity-fed extruders and a backward scheme for starve-fed extruders [166].

For classic extrusion (gravity-fed), the forward calculation (modeling) scheme is appropriate, whereas for non-classic extrusion (starve-fed), the backward (inverse) calculation (modeling) scheme is used (Figure 19).

In the classical extrusion process (with gravity feed, that is, without dosing), the extrusion capacity (process throughput) is not known, because it is not a process variable that can be set, but an output variable, i.e., it is not input data of the process, but output data. Therefore, the extrusion pressure can not be calculated, because it is a function of the polymer flow rate (process throughput). The extrusion process is defined by the characteristics of the screw and the die (dependence of the polymer flow rate on the pressure), the intersection point of which indicates the working conditions of the extruder (extrusion throughput and pressure), i.e., the so-called working point of the extruder. This working point is determined by iterative process calculations with a search for the convergence between the pressure rise in the screw and the pressure drop in the die. This modeling approach is presented, for example, in [128].

In the non-classical extrusion process (with starve feed, that is, with dosing), the extrusion capacity (process throughput) is known, because it is input data which is set by the extruder operator. However, the screw filling is not known. This is determined by iterative process calculations with a search for the convergence between the calculated polymer melt temperature and the polymer melting temperature at the point where melting is complete. This modeling approach is presented, for example, in [148].

The extrusion global model consists of serially connected elementary models of solid conveying, material fusion and melt conveying. When modeling, the lumped parameter approach is applied, that is, the locally constant material and process parameters are assumed, which is sufficient for engineering calculations [159]. The screw is divided into elementary units, and the process variables, for example, polymer temperature, polymer pressure, solids volume or screw filling, at the entrance to a given unit are equal to the variables at the outlet from the previous one, that is

π_i_input_ (ψ) = π_i−1_output_ (ψ), (1)

π_i_input_ (ψ) is the process variable (e.g., polymer pressure and temperature, solids volume, screw filling) at the entrance to i–unit, π_i−1_output_ (ψ) is the process variable at the outlet from (i − 1)–unit and ψ is the position of the unit Δψ along the length of screw channel.

#### 4.2.4. Slip Effects

One of the basic assumptions of fluid mechanics is the assumption of no slippage between the fluid and the surfaces that limit the flow space. And, this assumption is usually used in modeling the extrusion of plastics. However, it often happens that such slippage occurs, for example, in the processing of polymers such as (HDPE) and (PVC), filled plastics, elastomers or polymer composites and suspensions.

Mooney was the first who explored this phenomenon [170]. Subsequently, several studies were conducted to analyze the slip effects in SSE-machines by 1-D isothermal Newtonian analysis [171,172] as well as by 2-D analysis [173], and a reducing effect of wall slip on the energy consumption was observed. Viscoplastic flows with wall slippage in the barrel and screws were also analyzed in the SSE-machines [174,175] as well as the TSE-machines [117,176]. Very extensive research has been carried out by the group of Potente [116,171,177,178,179,180], who analyzed both analytically and numerically the fundamental process issues in SSE-machines, e.g., pressure vs. flow-rate relations and energy consumption. The slip effects were also investigated when modeling the flow in extrusion dies, e.g., [181,182,183].

A comprehensive 3-D non-Newtonian finite element flow analysis in the SSE-machine (using the Ansys-Polyflow system) was carried out by Wilczyński and Lewandowski [85,184,185], who developed the screw and die characteristics to implement them in a global process model. It can be clearly seen from these studies that the velocity distribution changes drastically and the pressure falls significantly with increasing slip, both in a die and an extruder.

It has now been settled that extrusion of polymers with wall slip causes a reduction in die pressure and a change in screw characteristics, which influences the extruder operating point and the results of a global modeling of extrusion. This requires the development of new models for both the screw and the die.

**Figure 20:**
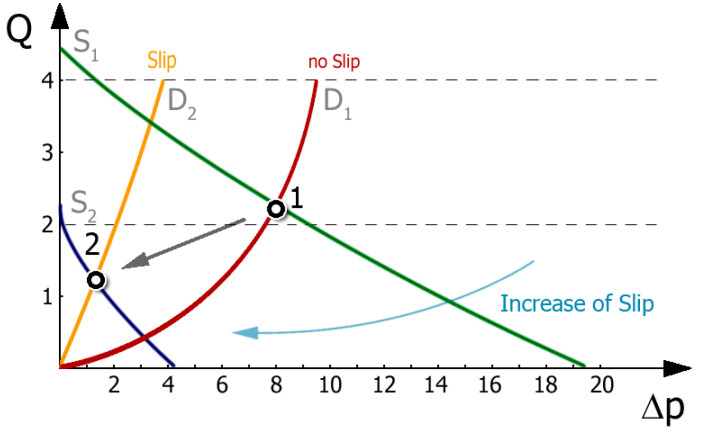
Extruder working chart (operating characteristics): Q—throughput (extrusion capacity), Δp—pressure, S_1_ (green line), S_2_ (violet line)—screw characteristics; D_1_ (bordo line), D_2_ (orange line)—die characteristics [167].

Extrusion is the process of forcing a polymer through a forming tool (extrusion die) and can be viewed as a link system of the extruder and the die. Extrusion can be considered in terms of the operating characteristic (working chart) of the extruder, which is defined by the screw characteristic and the die characteristic (Figure 20). The intersection of these characteristics indicates the working (operating) point of the extruder, and defines the process capacity (extrusion throughput) and extrusion pressure (die pressure).

Figure 20 shows the influence of slip conditions on the extruder operating point. The initial point (1), determined by the intersection of screw characteristics S_1_ (green line) and die characteristics D_1_ (bordo line), was obtained for low slip on the screw and no slip on the die. When slip on the die is allowed and slip on the screw increases, the operating point shifts towards a lower extrusion capacity and pressure, i.e., to point (2), determined by the intersection of screw characteristics S_2_ (violet line) and die characteristics D_2_ (orange line). It can be summarized that the slip on the screw and die has a significant influence on the position of the extruder operating point, i.e., on the extrusion capacity and pressure. As the slip on the screw grows, the extrusion capacity and pressure diminish, whereas as the slip on the die grows, the capacity grows and the pressure diminishes.

#### 4.2.5. Yield Stress Effects

Usually the materials flowing in extruders do not show yield stress; however, it often happens that yield stress occurs, for example, in the processing of filled plastics, polymer composites and suspensions, as well as paints, cosmetics and foodstuffs.

Bingham was the first who explored these materials (viscoplastics) [186]. Compared with general studies on the flows with yield stress [187,188] much fewer analyses have been carried out to these flows in extrusion, although several models have been developed for the flow in the SSE-machine [174,175], as well as the co-rotating TSE-machine [176].

A comprehensive 3-D non-Newtonian finite element (FEM) flow analysis of viscoplastics in the SSE-machine (using the Ansys-Polyflow system) was carried out by Wilczyński and Lewandowski [86,184,185], who developed the screw and die characteristics to implement them in a global process model. As a result of these studies, the effect of yield stress is clearly seen in the form of a flat region of the velocity distribution.

**Figure 21:**
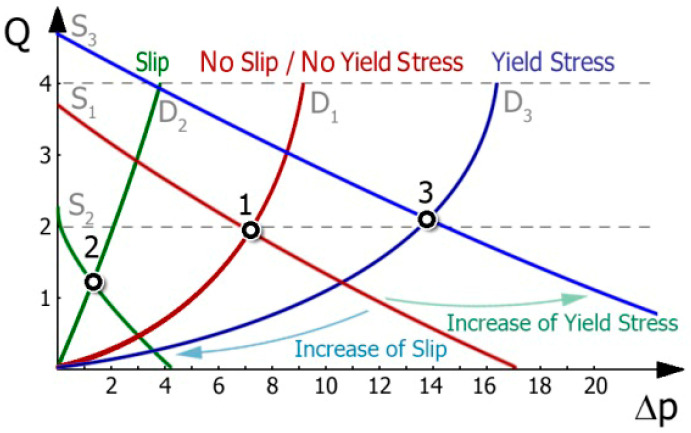
Extruder working chart (operating characteristics): Q—throughput (extrusion capacity), Δp—pressure, S_1_ (green line), S_2_ (violet line), S_3_ (blue line)—screw characteristics; D_1_ (bordo line), D_2_ (orange line), D_3_ (blue line)—die characteristics [184].

Figure 21 shows the influence of yield stress on the extruder working point. The initial point (1), determined by the intersection of screw characteristics S_1_ (bordo line) and die characteristics D_1_ (bordo line), was obtained for low-yield stress in the screw and no-yield stress in the die. When yield stress in the die is allowed and yield stress in the screw increases, the operating point shifts towards a higher extrusion capacity and pressure, i.e., to point (3), determined by the intersection of screw characteristics S_3_ (blue line) and die characteristics D_3_ (blue line). It can be summarized that the yield stress in the screw and die has a significant influence on the position of the extruder working point, i.e., on the extrusion capacity and pressure. As the yield stress in the screw increases, the extrusion capacity and pressure increase, whereas as the yield stress in the die increases, the capacity decreases and the pressure increases.

### 4.3. Polymer Injection Molding

Extrusion and injection molding are to some extent similar processes, with the main difference being that extrusion is a continuous one with material pushing through an opened die, whereas injection molding is a cyclic one with material injecting into a closed mold.

When modeling these important processes, a comprehensive approach must be used to include solids conveying, polymer fusion and melt conveying, both in the plasticating unit and in the die or mold. The model of material fusion provides the basis for building such global models. Conceptions developed for studying the extrusion process have been applied for modeling the injection molding process (IM-process).

Most of the studies for injection molding were devoted to the flow in injection molds, e.g., [189,190,191,192,193,194]. Currently, the most common injection molding simulation programs are MOLDFLOW [195], Moldex3D [196] and CADMOULD [197], e.g., recently used in [198,199]. Simulations were also performed using the CFD programs [200,201].

Research on the flow phenomena in the plasticating unit were much more limited. The first experiments in this area were carried out by Donovan et al. [202] who applied the “screw pulling-out technique”. The authors observed that when the fusion time (screw rotation phase) was a large fraction of the injection cycle time, the fusion was similar to the fusion in extruders, but when this time was a small fraction of the cycle time, the fusion was significantly different.

Very valuable studies on the flow phenomena in IM-machines were the visual ones [203,204,205]. As a result of these studies, it was found that at lower screw rotations (longer fusion time), more polymer was fused than at higher screw rotations (shorter fusion time). Moreover, at a lower stroke of fusion, fusion was faster, whereas at a higher stroke of fusion it was slower. And, fusion was faster with an increase of back pressure.

Donovan [206,207,208] built the first model of polymer fusion in an IM-machine. This required the use of an empirical parameter specific to a particular material within the range of process data being studied. Lipshitz et al. [209] developed a fusion model on the basis of analysis of physical phenomena in the IM-process. Furthermore, the issue of fusion has been discussed by others, for example, [210,211,212,213,214,215].

The original approach was presented by Bereaux et al. [216] who replaced the two-phase flow (solid and melt) with a single-phase flow (melt). Yung et al. [217,218,219] were the first to distinguish three stages of IM-process: fusion (rotating screw and moving backward), stopping (screw does not move) and injecting (screw moving forward).

A comprehensive model of polymer fusion in IM-machines was presented by Iwko and Steller [220,221,222], as well as by Fernandes et al. [223], Gaspar-Cunha et al. [224]. However, it is important to note, e.g., [166], that the only available computer program for simulating the IM-process is the PSI program from the University of Paderborn [225]. Recently, CoreTech presented the software [194] which allows for the study of the phenomena in the plasticating unit of the IM-machine; however, the computational models used there were not explained.

To sum up, it can be said that IM-models are based on the SSE-models and they do not have a solid and reliable experimental basis. There is a lack of research on the physical phenomena occurring in the IM-machines. The cause is that experiments with the “screw pulling-out technique” in IM-machines are enormously difficult. It is very difficult to quickly remove the screw from the machine to prevent the material from fusion.

That is why Wilczyński et al. [226] developed an original “screw pulling-out technique” for the IM-process, and conducted extensive experiments to reveal physical phenomena occurring in the IM-machines. It was obviously seen that starvation occurs when the screw moves forward and fills the mold. The level of starvation was dependent on the screw rotation, stroke of fusion and back pressure. Based on these observations, novel conceptions of IM-process modeling have been postulated.

**Figure 22:**
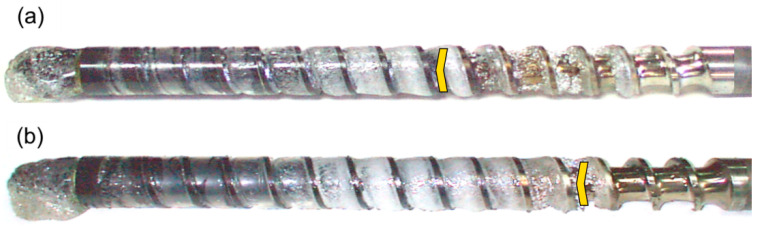
The screws from the IM-machine, (PS) injection: (**a**) N_1_ = 100 min^−1^, (**b**) N_2_ = 300 min^−1^, yellow mark—the beginning of starvation [226].

Examples of these investigations are depicted in Figure 22. It is seen that as screw rotation grows the polymer fusion occurs more slowly, and the starvation diminishes.

In general, the existing IM-process models discussed here are different from the SSE-models in that they include static and dynamic phases of fusion. However, the screw is assumed to be completely filled with polymer, as in the gravity-fed SSE-process (Figure 1), which is contradictory to our studies [226] in which starvation was observed, as in the starve-fed SSE-process (Figure 3).

Recently, Narowski and Wilczyński [227] stated that a comprehensive approach to modeling the IM-process is necessary, which includes the flow in the IM-machine and the injection mold. The resulting variables of the IM-process, e.g., polymer temperature and pressure, should be the input variables for the injection mold calculations. They also concluded that starvation observed in the IM-process [226] which has not been reported in the literature till now and which will significantly affect the IM-process modeling.

### 4.4. WPC Injection Molding

As indicated above, the modeling of the IM-process of polymers is rather uncritically based on the SSE-process models and does not have a solid experimental basis. Research on the IM-process of WPCs is very scarce and is limited only to the simulations of the flow in the mold.

Duretek et al. [228] indicated a great interest in the market for products made of WPCs as an alternative to pure thermoplastics in the injection molding process. And, they performed classical studies of WPCs’ IM-process, i.e., simulation and experimental validation, by visualization of the mold filling. They observed that in contrast to unfilled polymers the WPCs had reduced melt elasticity so that the fountain flow often did not develop. This results in irregular flow front shapes in the molded part, especially at high filler content. They concluded that the WPCs IM-process cannot be properly predicted using the existing 3-D simulation software since there are no appropriate rheological models of these materials available that take into account, for example, slip phenomena or material elasticity. They indicated the need for further studies on flow and filling behavior with WPCs with different wood contents to identify possible specific process phenomena.

Azaman et al. [229,230] performed a numerical analysis of the residual stress formation in the cavity of thin-walled injection molded parts and considered the residual stresses developed in the post-filling stage. The residual stress in the cavity is a fundamental step in the analysis because of the need to control the quality of molded parts to prevent shrinkage and deformation problems. The authors selected the optimal variables of the process and described the influence of these in the post-filling stage on the residual stress in the cavity, volumetric shrinkage and deformation properties.

Moreover, Rahman et al. [231] presented simulation analysis for injection molding of natural fiber composite (rice husk filled composite), and Zhan et al. [232] presented a very short review based on a few papers on some aspects of flow simulations in injection molds.

To summarize the above review on modeling the WPCs’ flows in polymer processing by extrusion and injection molding, it is worth mentioning several valuable papers on modeling other pressure flows. For example, Liese and Wünsch [233] considered different boundary conditions in their simulations, such as no slip, Navier slip and full slip, and Pashazadeh et al. [234] predicted the fiber orientation in extruded wood–plastic composites.

## 5. Summary and Conclusions

The presented paper can be summarized as follows.

Wood–plastic composites (WPCs) consist of thermoplastics and wood fillers. Polypropylene (PP), high density polyethylene (HDPE) and poly(vinyl chloride) (PVC) are the basic thermoplastics for WPCs. In addition to standard polymers, biodegradable materials can also be used as WPCs matrices, e.g., based on the polylactic acid (PLA), polyhydroxyalkanoates (PHA, PHB and PHBV), starch derivatives and cellulose, etc. Wood fillers are composed of natural polymers such as cellulose, etc., and have completely different properties to synthetic materials

The properties of WPCs are dependent on the kind of polymer matrix and wood filler and the processing conditions. They can be made better by adding compatibilizers or modifying the surface of fillers. The type of the compatibilization method used is important. Physical and chemical modifications can be used to improve the interfacial bonding between the filler and the matrix. Chemical processing is more complicated and has a large effect on the environment comparing with physical processing. But, unlike chemical processing, the equipment applied for physical modification is usually very expensive, e.g., plasma processing. So, it is necessary to focus on reducing the environmental pollution during chemical processing or the cost of physical processing.

Nanobiocomposites are very popular in the field of nanotechnology. These “materials of tomorrow” offer better properties, and can be applied in advanced uses. Different types of nanofillers can be used in WPCs, e.g., nanoclays are broadly applied since they are eco-friendly, easily available and cheap. Wood–plastic composites can be produced with the use of bio-based adhesives such as starch or lignin, which are environmentally friendly alternatives to conventional synthetic adhesives.

Wood–plastic composites have non-Newtonian, pseudoplastic and viscoelastic properties. Their viscosity diminishes with a growth of shear rate and temperature and grows with a growth of filler amount. Their elasticity diminishes with a growth of filler amount. These materials show a yield stress and wall slip when extruding. The slip velocity depends on the filler amount and shear rate. With a growth of shear rate, the slip velocity grows, leading to the plug flow. Moreover, increasing the filler amount promotes the plug flow.

The manufacturing process of wood–plastic composites includes two stages, that is, compounding and forming. The process of compounding is carried out to incorporate the fillers into a molten thermoplastic matrix. And, this composite is then formed into a product mainly by extrusion and injection molding. Manufacturing methods such as compression molding and pultrusion have certain limitations. The WPCs’ processing is significantly different from that of standard (neat) plastics due to the differences in the thermo-rheological properties of these materials and diverse structures, etc.

When modeling the important processes, extrusion and injection molding, a comprehensive approach must be used to include solid conveying, polymer fusion and melt flow. The models both for a plasticating unit and for an extrusion die or injection mold should be developed. The model of polymer fusion provides the basis for building such comprehensive models.

The only studies available till now on the flow mechanism of WPCs in the extrusion process were conducted by the authors, who showed that the composite conveying and fusion are significantly different from those of neat polymers and are dependent on the WF content, extrusion conditions and feeding mode. In the gravity-fed SSE-process, the classical 2-D fusion, i.e., the Tadmor mechanism, was seen for the composites with a lesser WF amount, up to 50%. However, it was not seen for the composites with more of a WF amount when 1-D fusion took place. In the starve-fed SSE-process with metered feeding, conductive fusion was seen in the not-fully-filled screw area, and classical 2-D fusion was observed in the completely filled area, which differs from the fusion of neat polymers in the starve-fed SSE-process, where dispersed fusion was seen.

On the basis of this research, the authors developed the only global WPCs extrusion models available to date for both gravity-fed and starve-fed extrusion. The models describe solid conveying, wood filler-dependent fusion and melt flow, and allow for the prediction of the process throughput or screw filling, the profiles’ pressure and temperature, fusion behavior and energy use. The models have been experimentally confirmed. So far, the models for the TSE-process, both co-rotating and counter-rotating, are still not developed. Also, flow mechanisms in regard to this have not been studied.

When global process modeling, calculation schemes should be used according to the extruder feeding mode. For classic extrusion (gravity-fed), the forward calculation (modeling) scheme is appropriate, whereas for non-classic extrusion (starve-fed), the backward (inverse) calculation (modeling) scheme should be used.

Wood–plastic composites show a yield stress and wall slip when extruding, which must be considered when modeling the process both in the screw and the die. As the slippage on the screw and barrel grows, the process throughput and pressure diminish, but as the slippage on the die grows, the process throughput grows and the pressure diminishes. As the yield stress in the screw grows, the process throughput and pressure grow, whereas as the yield stress in the die grows, the process throughput diminishes and the pressure grows. So far, there are no global models for WPCs’ extrusion that would simultaneously consider the slip and yield stress effects on the process.

The modeling of the IM-process is uncritically based on the SSE-process models and does not have a solid experimental basis. Research on the IM-process of WPCs is very scarce and is limited only to the simulations of the flow in the mold. However, these studies do not take into account the phenomena of wall slip and yield stress. To date, no studies on the flow of wood–plastic composites in IM-machines (solid conveying, fusion, etc.) have been carried out, neither theoretical nor experimental.

In general, the existing IM-models are different from the SSE-models in that they include static and dynamic phases of fusion. However, the screw is assumed to be completely filled with polymer, as in the gravity-fed SSE-process, which is not correct since an obvious starvation occurs in the IM-process, as in the starve-fed SSE-process. It is obvious that starvation occurs when the screw moves forward and fills the mold. The level of starvation is dependent on the screw rotation, stroke of fusion and back pressure.

A comprehensive approach to modeling the IM-process is necessary, which includes the flow in the IM-machine and the injection mold. The resulting variables of the IM-process, e.g., polymer temperature and pressure, should be the input variables for the injection mold calculations. Starvation observed in the IM-process which has not been reported in the literature till now will significantly affect the IM-process modeling.

When modeling the WPCs processing, certain material and process issues are of crucial importance.

In relation to material issues, it is worth noting that

- There is generally a lack of basic rheological data for wood–plastic composites in the available material databases;
- There is no data at all that takes into account slip and yield stress, and these data should be obtained in-house, preferably in on-line production conditions;
- There is generally no valid thermal data, e.g., melting or softening point or no-flow temperature, heat of fusion etc.

In relation to process issues, it is worth noting that they require a holistic approach. They comprise the following:

- Global modeling of extrusion (for gravity-fed and starve-fed, separately) including solid conveying, polymer fusion as well as melt flow in a plasticating unit and a die;
- Simultaneous modeling of fusion and melt flow using the CFD concept in extrusion;
- Simultaneous modeling of solid conveying, fusion and melt flow using the DEM/CFD concept in extrusion;
- Global modeling of injection molding which would include modeling of solid transport, polymer fusion and melt flow in the plasticating unit and the mold;
- Holistic modeling of gravity-fed and starve-fed extrusion with a smooth transition between these two feed modes;
- Holistic modeling of extrusion and injection molding with a smooth transition between these two techniques.

## Data Availability

No new data were created or analyzed in this study. Data sharing is not applicable to this article.
